# Glucagon-Like Peptide-1 Receptor Regulates Macrophage Migration in Monosodium Urate-Induced Peritoneal Inflammation

**DOI:** 10.3389/fimmu.2022.772446

**Published:** 2022-01-27

**Authors:** Jun Chen, Aihua Mei, Xinxin Liu, Zachary Braunstein, Yingying Wei, Biao Wang, Lihua Duan, Xiaoquan Rao, Sanjay Rajagopalan, Lingli Dong, Jixin Zhong

**Affiliations:** ^1^ Sinopharm Dongfeng General Hospital, Hubei University of Medicine, Hubei Key Laboratory of Wudang Local Chinese Medicine Research (Hubei University of Medicine), Shiyan, China; ^2^ Cardiovascular Research Institute, Case Western Reserve University, Cleveland, OH, United States; ^3^ Department of Rheumatology and Immunology, Tongji Hospital, Huazhong University of Science and Technology, Wuhan, China; ^4^ Department of Medicine, The Ohio State University Wexner Medical Center, Columbus, OH, United States; ^5^ Department of Biochemistry and Molecular Biology, School of Life Sciences, China Medical University, Shenyang, China; ^6^ Department of Cardiology, Tongji Hospital, Huazhong University of Science and Technology, Wuhan, China

**Keywords:** gout, GLP-1, glucagon-like peptide-1, macrophage, monosodium urate, inflammation

## Abstract

Glucagon-like peptide-1 (GLP-1) is an insulinotropic peptide that signals through the GLP-1 receptor (GLP-1R). GLP-1R, therefore, plays a critical role in diabetes and cardiovascular disease. Whether GLP-1R is involved in inflammatory disease such as gout remains unclear. Macrophages are critical effector cells in the pathogenesis of gout, a common form of inflammatory arthritis caused by the deposition of uric acid in joints. The expression of GLP-1R at the protein level is controversial due to the lack of specificity of existing antibodies against GLP-1R. Using a transgenic mouse model expressing enhanced green fluorescent protein (EGFP) under the control of GLP-1R promoter, here we confirmed the expression of GLP-1R by macrophages. M2 type macrophages and Ly6C^+^ macrophages expressed higher levels of GLP-1R, compared to their counterparts. GLP-1R deficient macrophages displayed a reduced the migratory ability and an enhanced expression of interleukin (IL)-6, while the expression of IL-1β was not affected. In monosodium urate (MSU) crystal-induced peritonitis, an experimental model of gout, the recruitment of macrophages, especially M2 macrophages, was significantly suppressed in GLP-1R knockout mice compared to wild-type mice. In conclusion, our data suggests that GLP-1R plays a critical role in macrophage migration in MSU-induced inflammation.

## Introduction

Glucagon-like peptide-1 (GLP-1) is an incretin peptide, secreted from intestinal enteroendocrine L cells (1). GLP-1 agonists have been increasingly used for the treatment of diabetes in clinical practice because of its insulinotropic beneficial effects against insulin resistance (2). The biological effects of GLP-1 are mediated by glucagon-like peptide-1 receptor (GLP-1R) (3), a seven-transmembrane G protein-coupled receptor family (4) that is expressed on many cell types, including macrophages (5–9). Translational and clinical studies have shown that GLP-1 and GLP-1R regulate glucose homeostasis and energy metabolism (10) and are involved in important physiological processes such as insulin release, β-cell proliferation, reduced glucagon release, delayed gastric emptying, and enhanced memory (11, 12). In addition, GLP-1 and GLP-1R have been implicated in immune regulation, by regulating macrophage polarization (13–16).

Macrophages are plastic cells of two main types, depending on their function: classically activated macrophages (M1 macrophages) and alternatively activated macrophages (M2 macrophages) (17). These two types of cells are interchangeable under specific signaling conditions. M1 macrophages can remove pathogenic microorganisms by producing primarily proinflammatory cytokines such as IL-6, IL-12, and tumor necrosis factor (TNF)-α. M2 macrophages ameliorate type 1 inflammatory responses and adaptive immunity by producing anti-inflammatory factors such as IL-1, IL-10, and transforming growth factor (TGF)-β, to promote type 2 immune responses and regulate tissue repair (17–19). Accumulating data indicate that macrophages play a critical role in the pathogenesis of inflammatory diseases, including gout.

As a common form of inflammatory arthritis caused by the deposition of uric acid in joints, gout can be very painful and debilitating (20, 21). Accumulating evidence suggests that disease activity is strongly associated with the activation of macrophages. The inflammatory response to monosodium urate (MSU) crystals triggers macrophage and neutrophil recruitment, leading to the symptoms of an acute gout attack. Intraperitoneal injection of MSU crystals in mice is used as an animal model of gout that can mimic acute episodes of gout (22). Following exposure to MSU, macrophages acquire a proinflammatory M1 phenotype instead of an anti-inflammatory M2 phenotype (23, 24). After M1 macrophage-induced endothelial cell damage in gout, M2 macrophages participate in tissue remodeling and repair (25, 26). However, little is currently known about the primary regulators that promote significant macrophage M1/M2 polarization in response to MSU stimulation in gout.

Given the important role of GLP-1/GLP-1R axis in regulating macrophage polarization (13–16), we hypothesize that it may regulate MSU-induced inflammation in gout. Here, we assessed the role of GLP-1R in MSU crystal-induced peritonitis by using wild-type (WT) and GLP-1R knockout (KO) mice. We found that MSU-induced inflammation was ameliorated in KO mice, and was characterized by decreased macrophage infiltration. Consistent with these findings, macrophages from GLP-1R KO mice exhibited reduced migratory ability *in vitro*. However, the number of neutrophils was not altered. Thus, the present results demonstrate that GLP-1R plays an important role in MSU-induced inflammation as demonstrated by the finding that GLP-1R deficiency reduced the migration of macrophages. Therefore, GLP-1R may serve as a potential therapeutic target for gout.

## Materials and Methods

### Animals

C57BL/6 mice (wild-type, WT), GLP-1R^Cre^, and ROSA26^EGFP^ mice in C57BL/6 background were purchased from the Jackson Laboratory. GLP-1R knockout mice (KO) were generated by crossing CMV-Cre mice with GLP-1R^flox/flox^ mice (27), a generous gift from Dr. Randy Seeley’s lab. GLP-1R^EGFP^ reporter mice were generated by crossing GLP-1R^Cre^ with ROSA26^EGFP^ mice. The mice were housed in the specific pathogen- free animal facility at Case Western Reserve University and Tongji Hospital. All experimental procedures involving mice were approved by the Institutional Animal Care and Use Committees (IACUCs) at Case Western Reserve University and Tongji Hospital affiliated to Huazhong University of Science and Technology.

### Establishment of Acute Gout Animals Model

Male mice (7-9 weeks of age) were administered an intraperitoneal (i.p.) injection of 3 mg MSU (*In vivo*Gen, US). Sixteen hours after i.p. injection of MSU, peritoneal exudate cells (PECs) were harvested by peritoneal lavage and subjected to flow cytometric analysis.

### Flow Cytometry Analysis

The PECs were incubated with fluorochrome-conjugated monoclonal antibodies. Antibodies used for flow cytometry were as follows: Pacific blue-labeled anti-mouse F4/80, PE-labeled anti-mouse CD11c, BV605-labeled anti-mouse CD206, APC-labeled anti-mouse CD11b, PE/CY7-labeled anti-mouse Ly6G (Gr-1), PE-labeled anti-mouse Ly6C. All antibodies were purchased from Biolegend (San Diego, CA, US).

### Quantitative Real Time RT-PCR

Total RNA was extracted from PECs by TRIzol^®^ Reagent (Invitrogen, Carlsbad, CA) as instructed. Reverse transcription of total RNA was performed using cDNA synthesis kit (ABI, ON, Canada). The primers for β-actin, IL-1β, IL-6, MIF, NLRP3, and GLP-1R gene are listed below: β-actin, forward: ACC TTC TAC AAT GAG CTG CG, reverse: CTG GAT GGC TAC GTA CAT GG; IL-1β, forward, ACG GAC CCC AAA AGA TGA AG, reverse: TTC TCC ACA GCC ACA ATG AG; IL-6, forward: ACA AAG CCA GAG TCC TTC AGA GAG, reverse: TTG GAT GGT CTT GGT CCT TAG CCA; MIF, forward: GAG GGG TTT CTG TCG GAG C, reverse: GTT CGT GCC GCT AAA AGT CA; NLRP3, forward: CTC CAA CCA TTC TCT GAC CAG, reverse: ACA GAT TGA AGT AAG GCC GG; GLP-1R, forward: TGA CCG ACT GTT TGT TCT CTT G, reverse: CCA ACT TAT GAC CTT CTG GTG AC. The expression of target genes was normalized to β-actin.

### Macrophage Migration Assay

Mature macrophages from GLP-1R^EGFP^, WT and KO in Dulbecco modified Eagle medium (DMEM) with 10% Fetal Bovine Serum were placed in the insert of a 5-μm Transwell^®^ plate (Sigma-Aldrich) at the concentration of 1×10^6^ cells/mL (100 μl/well). The lower chamber was filled with 600 μl DMEM with 10% Fetal Bovine Serum and MIP-2 (200 ng/ml, Biolegend). After 4 hours of incubation at 37°C, cells remained in the insert or migrated to the bottom wells were collected and detected by flow cytometry.

### Crystal Violet Staining

Crystal Violet staining of the Transwell^®^ membrane was performed to evaluate the number of transmigrating through the membrane. In brief, medium was removed from the Transwell^®^ plate and the membranes in the insert were gently rinsed with 1x PBS. Cells remained on the membrane were stained with 0.2% Crystal Violet for 10 min and wash thoroughly with distilled water until no dye is washed off. After air dry, the membrane was then observed and images were captured under an optical microscope. Then the crystal violet dye on membranes was dissolved with 33% acetic acid. The optical density (OD) of the solution at 570 nm, which is directly proportional to the number of cells remained on the membrane, was then detected on a microplate reader.

### Statistical Analysis

Data is presented as mean ± standard error of mean (SEM). Group comparisons were performed using Student’s t-test or analysis of variance (ANOVA) by GraphPad Prism software. p-values (two-tailed) below 0.05 were considered as statistically significant.

## Results

### Administration of a GLP-1R Agonist Does Not Affect Macrophage Expression of GLP-1R

**Figure 1:**
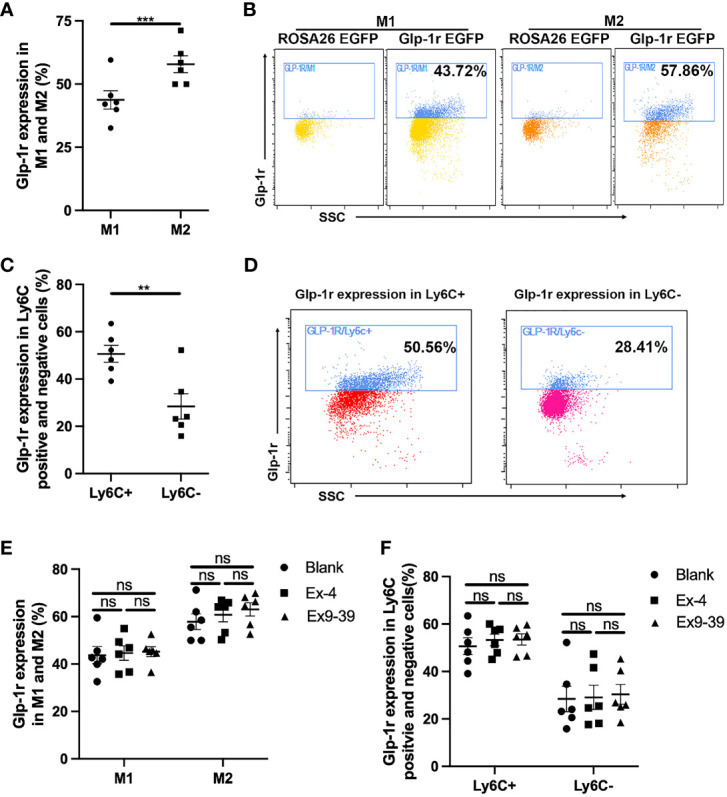
GLP-1R expression is not affected by agonist. PECs were collected from GLP-1R^EGFP^ mice. GLP-1R agonist Ex-4 was added to the medium with or without GLP-1R antagonist Ex 9-39. After 24 h, cells were harvested and stained with Pacific Blue-labeled anti-mouse F4/80, PE-labeled anti-mouse CD11c or PE-labeled anti-mouse Ly6C, and APC-labeled anti-mouse CD206. **(A, B)** Frequency of GLP-1R positive cells in M1 and M2. **(C, D)** Frequency of GLP-1R positive cells is higher in Ly6C+ subsets than that in Ly6C-. **(E, F)** GLP-1R agonist does not affect the frequency of GLP-1R positive macrophages. Data is presented as mean ± SEM. N=6/group. The results shown are from one of three independent experiments. **p < 0.01, ***p < 0.001. ns, not significant.

GLP-1R is widely distributed throughout the body including macrophages. We first examined the expression of GLP-1R on the surface of macrophages and whether its expression was affected by exposure to Ex-4 and Ex-9-39, as a GLP-1R agonist and antagonist, respectively (13). We found that GLP-1R was widely expressed in macrophages. As shown in **Figures 1A, B**, the expression of GLP-1R was greater in M2 macrophages than in M1 macrophages. The expression of GLP-1R also differed between Ly6C-positive and Ly6C-negative cells, with greater expressivity in Ly6C-positive cells (**Figures 1C, D**). Interestingly, we found that exposure to either Ex-4 or Ex-9-39 did not affect GLP-1R expression in M1 or M2 macrophages (**Figures 1E, F**). Collectively, this data indicates that GLP-1R is differentially expressed in different macrophage types, but its expression was not affected by exposure to an agonist or antagonist.

### GLP-1R Enhances the Migratory Ability of Macrophages

**Figure 2:**
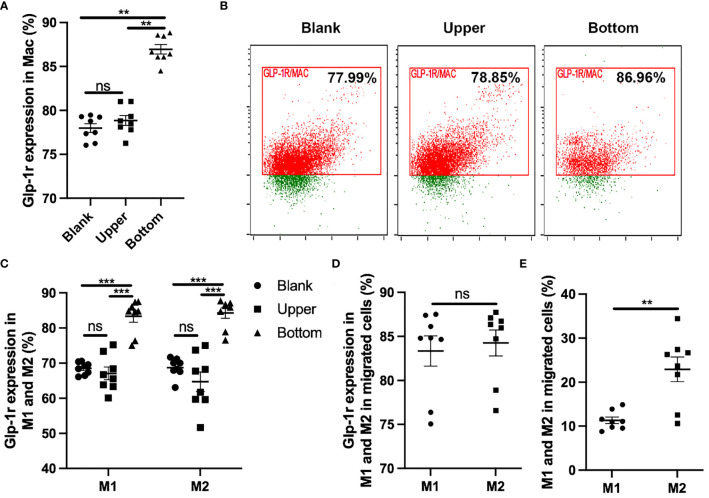
GLP-1R can enhance the migratory ability of macrophage. PECs were collected from GFP-GLP-1R mice and 1×10^6^ cells were seeded into the upper chamber. 600 μl DMEM with 10% FBS and MIP-2 (200ng/ml, PeproTech) was added to the lower chamber. After 4 h, cells were collected from the upper and lower chambers respectively, and labeled with PB anti-mouse F4/80, PE anti-mouse CD11c, APC anti-mouse CD206. **(A, B)** GLP-1R expression on the migrated macrophage is higher than the blank and upper. **(C)** GLP-1R expression on migrated M1 and M2 are higher than the blank and upper. **(D)** Frequency of GLP-1R positive cells in migrated M1 and M2 macrophages. **(E)** The ratio of M2 is higher than M1 in migrated cells. Blank group indicates the cells cultured in a 12-well plate in the presence of 200ng/ml for the same period (4h). Data is presented as mean ± SEM. N=8/group. The results shown are from one of three independent experiments. **p < 0.01, ***p < 0.001. ns, not significant.

**Figure 3:**
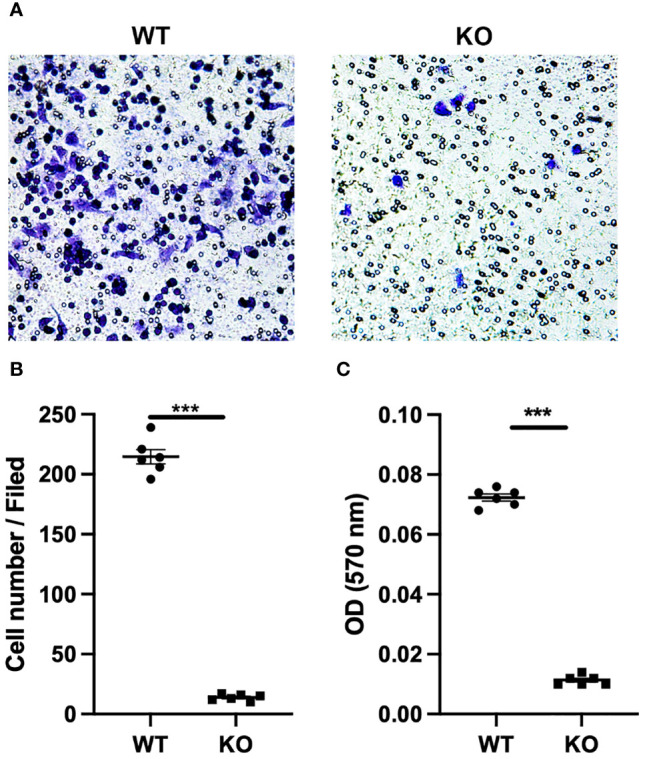
GLP-1R knockout affects the peritoneal exudate cells migration. PECs were collected from untreated WT and GLP-1R KO mice. 600 μl of DMEM with 10% FBS and MIP-2 (200ng/ml, PeproTech) was added to the lower chamber. After incubation for 4h in 37°C, migration was assessed by fixing the cells at the bottom of the transwell membrane with methanol followed by staining with crystal violet (0.1%, Sigma-Aldrich). **(A)** Representative pictures of the stained migrated cells at the bottom of the Transwell^®^ membrane were taken using an inverted microscope (n=6). **(B)** Quantification of the number of PECs at the bottom of the transwell membrane(n=6). **(C)** Crystal violet was resolved with 33% acetic acid and the absorbance was measured by microplate reader at 570 nm (n=6). After subtracting the optical density (OD) value of blank well, the ODs of WT and KO wells are presented. Data is presented as mean ± SEM. The results shown are from one of three independent experiments. ***p < 0.001. ns, not significant.

Migratory ability plays a key role in the function of macrophages. Different macrophage types exert different biological effects and show significant differences in migratory ability. Here, we performed Transwell assays to determine the effects of GLP-1R expression on the migratory ability of macrophages. We first found that GLP-1R expression was greater in the migrated macrophages than in cells in the upper chamber (**Figures 2A, B**), with similar results for M1 and M2 macrophages (**Figure 2C**). GLP-1R expressions in migrated M2 and migrated M1 macrophages were at a similar level (**Figure 2D**). In addition, the frequencies of both GLP-1R^+^ M1 and M2 were higher in the bottom well compared to those in the insert (**Figure 2C**). These results suggested that most of the migrated macrophages including both M1 and M2 expressed GLP-1R. The proportion of M2 macrophages was significantly greater than that of M1 macrophages among the migrated cells (**Figure 2E**). We also used GLP-1R KO mice to further investigate the effects of GLP-1R on macrophage migration. In this experiment, PECs were collected from WT and GLP-1R KO mice, and seeded into the upper chamber. Transwell membranes were stained with crystal violet and the migrated cells were examined under an inverted microscope. The reduced number of cells on the membrane reflects the fact that GLP-1R KO inhibits the migratory ability of macrophages (**Figures 3A, B**). The OD value of acetic acid eluent further confirmed the above results (**Figure 3C**). Collectively, this data demonstrates that GLP-1R enhances the migratory ability of macrophages.

### GLP-1R Affects IL-6 Production, but Does Not Affect IL-1β

**Figure 4:**
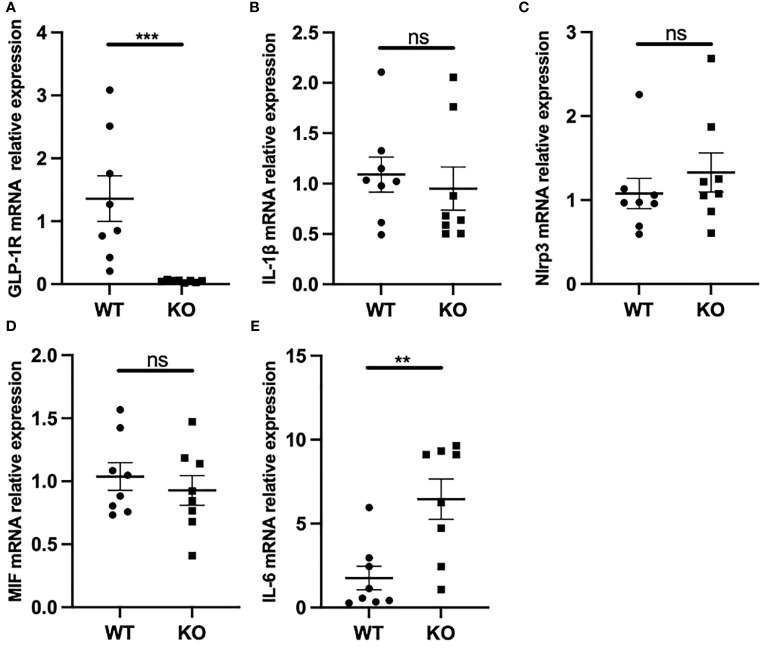
GLP-1R had no effect on IL-1β but affected IL-6 production. WT and GLP-1R KO mice were i.p. injected with MSU. After 16 hours, the total PECs were harvested. **(A-E)** GLP-1R, IL-1β, IL-6, Nlrp3 and MIF mRNA expression of macrophages was analyzed by real-time PCR. Data is presented as mean ± SEM (n=8-10/group). The results shown are from one of three independent experiments. **p < 0.01, ***p < 0.001. ns, not significant.

Macrophages can phagocytose MSU crystals, leading to the release of proinflammatory cytokines such as IL-1β, IL-6, and TNF-α. It has been shown that IL-1β produced by macrophages is a key inflammatory mediator in MSU crystal-induced inflammation. Furthermore, MSU induces massive neutrophil infiltration in WT mice, an effect that was suppressed in IL-1 receptor KO mice (9). Because we observed a marked change in the migratory ability of macrophages in GLP-1R KO mice, we next assessed the mRNA expressions of several classic proinflammatory genes in macrophages. As expected, GLP-1R expression was nearly undetectable in macrophages from GLP-1R KO mice (**Figure 4A**). Unexpectedly, IL-1β mRNA expression was not significantly different between the WT and KO mice (**Figure 4B**). Similar results were seen in *in vitro* experiments using bone marrow-derived macrophages (**Figure S1**). IL-1β maturation requires inflammasome activation (28). Similar to the results for IL-1β, GLP-1R KO did not affect the expression of NLRP3, the rate-limiting molecule for NLRP3 inflammasome (**Figure 4C**). Furthermore, the expression of macrophage migration inhibitory factor (MIF) was similar between WT and KO mice (**Figure 4D**). Interestingly, there was an increase in IL-6 expression in KO mice (**Figure 4E**).

### Macrophage Recruitment Is Reduced in GLP-1R KO Mice

**Figure 5:**
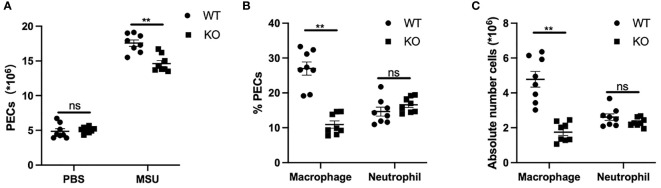
Decreased macrophage recruitment in GLP-1R KO mice after stimulation. WT and GLP-1R KO mice were treated with MSU to induce peritonitis. After 16 hours, the total PECs were harvested. **(A)** Quantitative analysis of total number of PECs. **(B)** The PECs were stained with anti-F4/80, anti-CD11b, and anti-Gr-1 antibodies. CD11b+ cells were gated for the analysis of neutrophils (CD11b+Gr-1+F4/80−) and macrophages (CD11b+Gr-1-F4/80+). Histogram showing the ratio of neutrophils and macrophages in total CD11b+ cells. **(C)** Absolute number of neutrophils and macrophages in the peritoneal exudate cells. Data is presented as mean ± SEM (n=8-10/group). The results shown are from one of three independent experiments. **p < 0.01, ns, not significant.

To further explore the effects of GLP-1R on the migratory ability of macrophages *in vivo*, we used MSU-induced peritonitis in mice as an animal model of gout. Six hours after MSU challenge, the total PECs were harvested for flow cytometric analysis. Compared with WT mice, the number of inflammatory cells in the peritoneal cavity was markedly reduced in GLP-1R KO mice (**Figure 5A**). The proportion of macrophages, defined as CD11b^+^F4/80^+^ cells, was significantly reduced in the peritoneal cavity of GLP-1R KO mice. However, there was no difference in the proportion of neutrophils (CD11b^+^Ly6G^+^F4/80^−^ cells) in the peritoneal cavity (**Figure 5B**). To confirm the role of GLP-1R in MSU-induced macrophage and neutrophil infiltration, we determined the absolute number of macrophages and neutrophils in the peritoneal cavity. In GLP-1R KO mice, the absolute number of macrophages was considerably decreased, when compared to WT mice, but the absolute number of neutrophils was similar between WT and GLP-1R KO mice (**Figure 5C**). To exclude the potential involvement of chemokines in this process, we examined the levels of CCL-2 and MIP-2 in the peritoneal flush fluid and no significant differences were observed between WT and KO mice (**Figure S2**), suggesting that the reduced macrophage migration in GLP-1R KO is independent of chemokine production.

### MSU-Induced Inflammation Significantly Increases the Proportion of M1 Macrophages but Not M2 Macrophages

**Figure 6:**
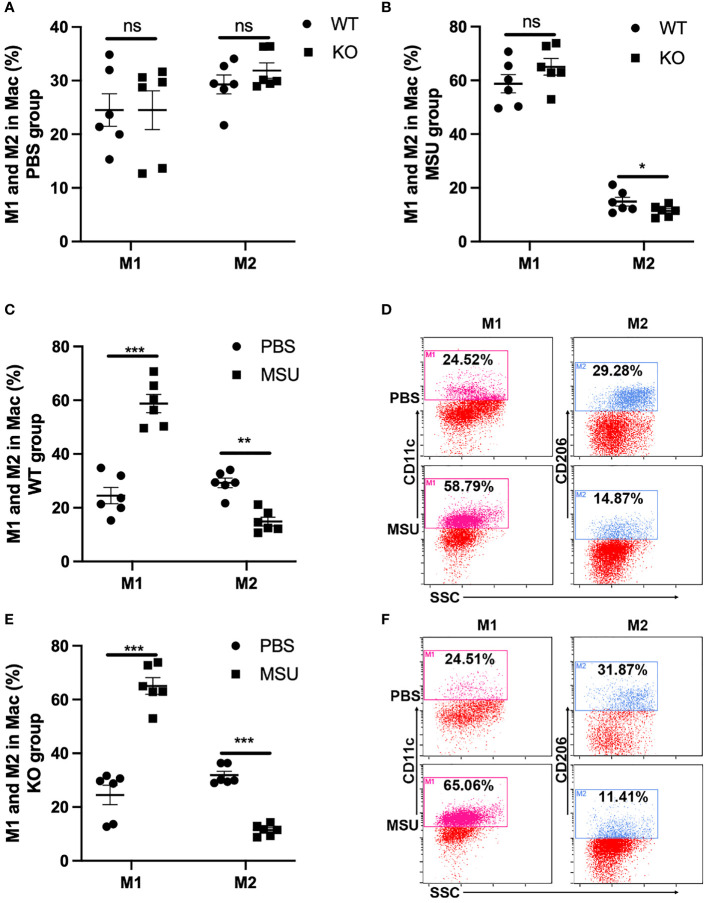
MSU-induced inflammation significantly increases the proportion of M1. WT and GLP-1R KO mice were treated with MSU to induce peritonitis. After 16 hours, the total PECs were harvested. **(A)** There is no difference in the proportion of M1 and M2 macrophages between WT and KO mice in PBS group. **(B)** There is no difference in the proportion of M1 macrophage but there is a difference in M2 macrophages between WT and KO mice after treatment with MSU. **(C–F)** MSU-induced inflammation can lead to the proportional change of M1 and M2 macrophages in both WT and KO group. Data is presented as mean ± SEM (n=8-10/group). The results shown are from one of three independent experiments. *p < 0.05, **p < 0.01, ***p < 0.001. ns, not significant.

Macrophages acquire a proinflammatory M1 phenotype, instead of an anti-inflammatory M2 phenotype, in response to MSU (23, 24). Of note, the proportion of M1 and M2 macrophages was similar between WT and KO mice in PBS group (**Figure 6A**). In the MSU group, the proportion of M2 macrophages was lower in KO mice than in WT, but there was no difference in the proportion of M1 macrophages (**Figure 6B**). As expected, administration of MSU significantly increased the proportion of M1 macrophages in the WT and KO mice, and the proportion of M2 macrophages decreased in both types of mice (**Figures 6C–F**).

## Discussion

The expression, especially at protein level, of GLP-1R has remained controversial in many cell types, including macrophages (9). By crossing GLP-1R^Cre^ mice with ROSA26^tdRFP^ mice, Richards et al. generated a transgenic GLP-1R reporter mouse model that expressed tdRFP, a red fluorescent protein, in GLP-1R-expressing cells. The authors characterized the GLP-1R expression profile in pancreatic cells, neurons, and in the cardiovascular system (9). However, the expression GLP-1R in immune cells, including macrophages, has not been determined. In this study, we generated a GLP-1R reporter mouse model using the similar strategy and confirmed the expression of GLP-1R in different subpopulations of macrophages. In this study, we found that the frequency of GLP-1R^+^ cells was higher in M2 subpopulation compared to M1. However, this phenomenon was not observed in the cells used in Transwell^®^ assay. This is probably due the stimulation with chemokine MIP-1 may affect the expression of GLP-1R or M1/M2 ratio, which requires future investigations. We observed that macrophage migratory ability is regulated by GLP-1R. GLP-1R deficiency did not affect IL-1β production or the migratory ability of neutrophils, but it did increase IL-6 production and macrophage polarization. These changes possibly led to the change in the migratory ability of the macrophages.

Exendin-4, a long-acting analog of GLP-1, is an agonist of GLP-1R that inhibits the adhesion of mouse macrophages and bladder cells (28). Exendin-4 transgenic mice were characterized by extensive lymphocyte infiltration, with increased numbers of CD4^+^ T cells and CD8^+^ T cells in the liver and kidney, and increased B220^+^ cells in the pancreas and liver. This suggests that exendin-4 promotes tissue infiltration of immune cells (29). Shirazi et al. (30) injected exendin-4 into the lateral ventricle of type 2 diabetic rats and observed increased expression of IL-6 and IL-1 in the hypothalamus and hindbrain, and increases in phosphorylated signal transducer and activator of transcription-3 (STAT3) and suppressor of cytokine signaling-1 (SOCS1). These results suggest that exendin-4 activates the STAT3/SOCS1 pathway in the hypothalamus and hindbrain, increases IL-6 and IL-1 levels, and promotes inflammatory responses. In the present study, we demonstrated for the first time a significant decrease in the number of inflammatory cells in the peritoneal cavity of GLP-1R KO mice treated with MSU. GLP-1R expression was not affected by treatment with a GLP-1R agonist. IL-1β is believed to be a critical proinflammatory cytokine in the pathogenesis of gout, and exerts a wide range of systemic and local effects (22). In humans, IL-1β production by resident macrophages is stimulated by MSU crystals, and plays a crucial role in the pathogenesis of gout (31). In a previous study, the exposure of macrophages to lipopolysaccharide caused a release of catecholamines, and the production of catecholamines by macrophages during inflammation was found to involve an autocrine/paracrine self-regulatory mechanism (32). Here, we did not observe a change in macrophage IL-1β production, but IL-6 production was increased in KO mice. This data suggests that the increased production of cytokines induced by catecholamines is independent of GLP-1R.

The GLP-1/GLP-1R signaling pathway is associated with PI3K and cAMP (12). GLP-1 exerts its hypoglycemic, cardiovascular protective, neuroprotective, and immune regulatory effects *via* GLP-1R (13, 33, 34). Upon activation, GLP-1R promotes cAMP production by adenylate cyclase, while enhancing glucose-stimulated insulin secretion (35, 36). Shiraishi et al. (14) showed that stimulating RAW264.7 human monocyte-derived macrophage cells with GLP-1 *in vitro* resulted in upregulated surface expression of CD163 and CD204, increased IL-10 secretion, and activated the STAT3 pathway, one of the main signaling pathways related to macrophage polarization towards the M2 phenotype (37–39). Macrophage polarization is a highly plastic physiological process that responds to a variety of factors, by changing macrophage phenotype and function (40). Furthermore, indirect effects of IL-6 on macrophage migration have been reported (41). In our study, we observed a significant relationship between GLP-1R and the migratory ability of macrophages, which may be achieved by regulating the polarization of macrophages by GLP-1R and the effects of IL-6. GLP-1R supports the migratory ability of macrophage. The number of recruited macrophages was significantly reduced in GLP-1R KO mice treated with MSU, as compared with WT mice. However, it is noteworthy that GLP-1R was globally knocked out in the KO mice used in our study. Although the effect of GLP-1R deficiency on the migratory ability of macrophage was confirmed in the *in vitro* Transwell^®^ migration assay, whether GLP-1R deficiency in other types of cells may contribute to the reduction of macrophage in MSU-treated GLP-1R KO mice need further investigation. In summary, our findings indicate that GLP-1R plays a critical role in the development of gout by promoting macrophage recruitment into sites of inflammation.

## Data Availability Statement

The original contributions presented in the study are included in the article/**Supplementary Material**. Further inquiries can be directed to the corresponding authors.

## Ethics Statement

The animal study was reviewed and approved by Institutional Animal Care and Use Committees (IACUCs) at Case Western Reserve University.

## Author Contributions

JZ conceived the project, designed the experiments, and revised the manuscript. JC and AM were responsible for design and performance of experiments, analyzed data and wrote the paper. BW, LihD, XL, ZB, YW, XR, LinD, and SR contributed to data analysis and revision. All the authors read, critically revised, and agreed to be accountable for the content of manuscript.

## Funding

This work was supported by grants from National Natural Science Foundation of China (81974254, 31870906, 82170470, 81671544, 81871286), National Institutes of Health (R01HL142643, R03DK119680), American Heart Association (17GRNT33670485), American Diabetes Association (1-19-JDF-117), American Association of Immunologists (CIIF-8745), the Faculty Development Grants from Hubei University of Medicine (2018QDJZR04), the Foundation of Health Commission of Hubei (WJ2021M061), the Natural Science Foundation of the Bureau of Science and Technology of Shiyan City (grant no. 21Y71), and Hubei Key Laboratory of Wudang Local Chinese Medicine Research (Hubei University of Medicine) (Grant No.WDCM2020010).

## Conflict of Interest

The authors declare that the research was conducted in the absence of any commercial or financial relationships that could be construed as a potential conflict of interest.

## Publisher’s Note

All claims expressed in this article are solely those of the authors and do not necessarily represent those of their affiliated organizations, or those of the publisher, the editors and the reviewers. Any product that may be evaluated in this article, or claim that may be made by its manufacturer, is not guaranteed or endorsed by the publisher.
