# Corrigendum: Glucagon-Like Peptide-1 Receptor Regulates Macrophage Migration in Monosodium Urate-Induced Peritoneal Inflammation

**DOI:** 10.3389/fimmu.2022.876934

**Published:** 2022-03-03

**Authors:** Jun Chen, Aihua Mei, Xinxin Liu, Zachary Braunstein, Yingying Wei, Biao Wang, Lihua Duan, Xiaoquan Rao, Sanjay Rajagopalan, Lingli Dong, Jixin Zhong

**Affiliations:** ^1^Sinopharm Dongfeng General Hospital, Hubei University of Medicine, Hubei Key Laboratory of Wudang Local Chinese Medicine Research (Hubei University of Medicine), Shiyan, China; ^2^Cardiovascular Research Institute, Case Western Reserve University, Cleveland, OH, United States; ^3^Department of Rheumatology and Immunology, Tongji Hospital, Huazhong University of Science and Technology, Wuhan, China; ^4^Department of Medicine, The Ohio State University Wexner Medical Center, Columbus, OH, United States; ^5^Department of Biochemistry and Molecular Biology, School of Life Sciences, China Medical University, Shenyang, China; ^6^Department of Cardiology, Tongji Hospital, Huazhong University of Science and Technology, Wuhan, China

**Keywords:** gout, GLP-1, glucagon-like peptide-1, macrophage, monosodium urate, inflammation

In the original article, there was an error in the statement of the source of GLP-1R knockout mice (KO).

A correction has been made to **Materials and Methods**, *“Animals”*:

C57BL/6 mice (wild-type, WT), GLP-1R^Cre^, and ROSA26^EGFP^ mice in C57BL/6 background were purchased from the Jackson Laboratory. GLP-1R knockout mice (KO) were generated by crossing CMV-Cre mice with GLP-1R^flox/flox^ mice (1), a generous gift from Dr. Randy Seeley’s lab. GLP-1R^EGFP^ reporter mice were generated by crossing GLP-1R^Cre^ with ROSA26^EGFP^ mice.

The authors apologize for this error and state that this does not change the scientific conclusions of the article in any way. The original article has been updated.

## Publisher’s Note

All claims expressed in this article are solely those of the authors and do not necessarily represent those of their affiliated organizations, or those of the publisher, the editors and the reviewers. Any product that may be evaluated in this article, or claim that may be made by its manufacturer, is not guaranteed or endorsed by the publisher.
