# Supplementary material for: Glucagon-Like Peptide-1 Receptor Regulates Macrophage Migration in Monosodium Urate-Induced Peritoneal Inflammation
Source: Front Immunol. 2022 Jan 27;13:772446. doi: 10.3389/fimmu.2022.772446 (PMC8828485; doi:10.3389/fimmu.2022.772446)
Supplement: Supplementary file 1 [file DataSheet_1.docx]

**Supplementary Figures and Figure Legends:**


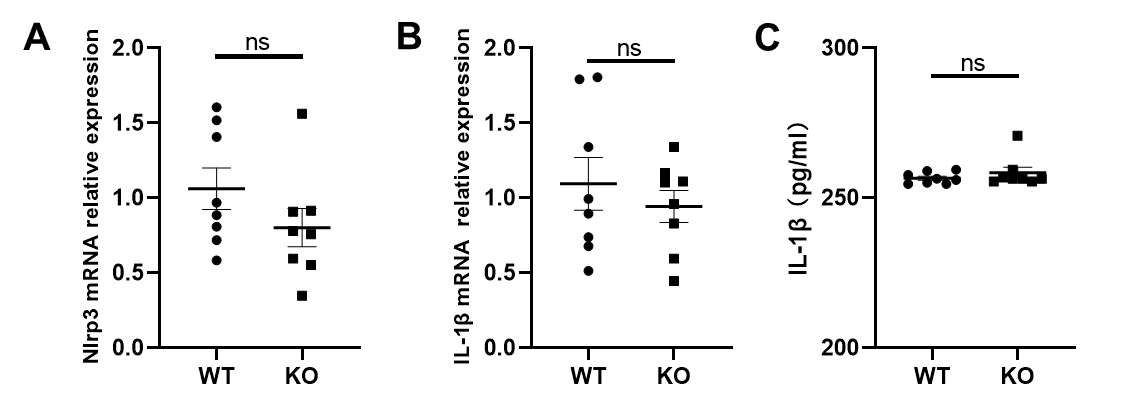


**Figure S1 GLP-1R had no effect on inflammasome activation.** Bone marrow cells isolated from WT and GLP-1R KO mice were cultured in the presence of 10 ng/mL M-CSF for 5 days, followed by priming with 100ng/mL LPS for 2h and MSU stimulation for 16h. Cells were harvest for realtime PCR detection of Nlrp3 (**A**) and Il1b (**B**) mRNA expression. Culture supernatant was used for the ELISA detection of IL-1β (**C**). n=8/group). ns, not significant.


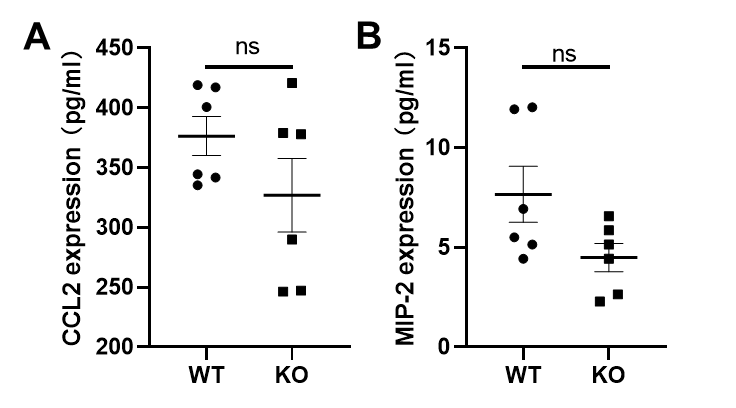


**Figure S2 GLP-1R deficiency did not affect production of macrophage chemokines CCL2 and MIP-2.** WT and GLP-1R KO mice were i.p. injected with MSU. After 16 hours, peritoneal cavity was flushed with 4 mL PBS. After centrifugation, the supernatant was collected for the ELISA detection of macrophage chemokines CCL2 (**A**) and MIP-2 (**B**). n=6/group). ns, not significant.
